# Elevated Plasma CXCL8 Concentrations in Significant Fibrosis but Not in Subclinical Rejection After Adult Liver Transplantation

**DOI:** 10.1097/TXD.0000000000001592

**Published:** 2024-02-21

**Authors:** Alejandro Campos-Murguia, Katharina Luise Hupa-Breier, Björn Hartleben, Heiner Wedemeyer, Richard Taubert, Bastian Engel

**Affiliations:** 1 Department of Gastroenterology, Hepatology, Infectious Diseases and Endocrinology, Hannover Medical School, Hannover, Germany.; 2 Institute for Pathology, Hannover Medical School, Hannover, Germany.

## Abstract

**Background.:**

The noninvasive detection of subclinical graft injury including subclinical T cell–mediated rejection (subTCMR) is one of the unresolved challenges after liver transplantation. Recently, serum C-X-C motif chemokine ligand 8 (CXCL8) was proposed as a highly accurate marker of subTCMR in pediatric liver transplant recipients. We aimed to evaluate the accuracy of the quantification of this chemokine for predicting subTCMR in adult liver transplant recipients, as well as its capacity to classify patients who could benefit from immunosuppression reduction.

**Methods.:**

Plasma CXCL8 concentrations were measured retrospectively in a prospectively collected cohort of adult liver transplant recipients with well-characterized histologic phenotypes.

**Results.:**

In total, 78 patients were included. Median plasma CXCL8 concentrations did not differ (*P* = 0.24) between patients without histological evidence of rejection (3.6 [0.4–22.0] pg/mL), subTCMR (11.5 [0.4–41.0] pg/mL), clinical TCMR (9.4 [0.4–40.5] pg/mL), and other etiologies of graft injury (8.7 [0.4–31.2] pg/mL). Likewise, plasma CXCL8 concentrations did not discriminate between patients within and outside histologic criteria for immunosuppression reduction that were proposed by the 2016 Banff Working Group on Liver Allograft Pathology (cutoff: 10.9 pg/mL, sensitivity: 0.48, and specificity: 0.79). Furthermore, weak correlation was found between plasma CXCL8 and alanine aminotransferase and aspartate aminotransferase (Spearman ρ = 0.18 and 0.25). Patients with significant fibrosis (17.8 [0.4–40.5] pg/mL) showed higher plasma CXCL8 concentrations than patients without fibrosis (8.2 [0.4–41.0] pg/mL; *P* = 0.05).

**Conclusions.:**

Plasma CXCL8 concentrations are not predictive of subclinical graft injury or of histological criteria for the minimization of immunosuppression in adult liver transplant recipients.

## Graphical abstract

**Figure:**
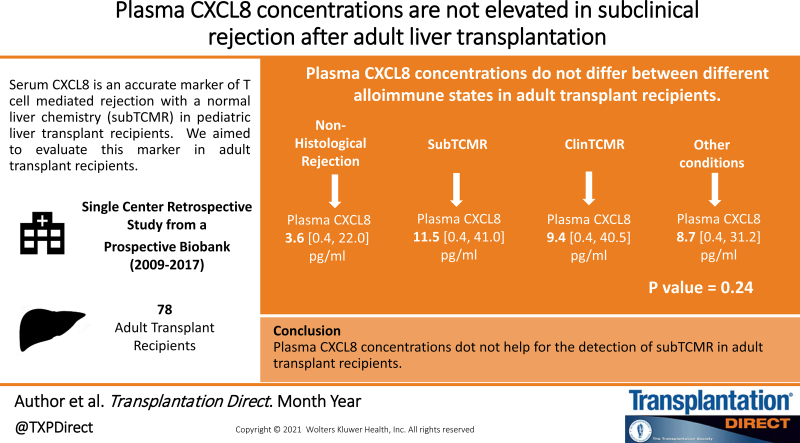

Over the past decades, there has been no improvement in the long-term survival of adult liver transplant recipients (aLTR).^1^ One of the factors responsible for this stagnation is the long-term side effects of immunosuppression (IS).^1^ Personalized IS using protocol biopsies based on the Banff Mini histologic criteria for IS reduction has been shown to be safe and effective in improving outcomes such as preservation of kidney function while avoiding graft rejection.^2,3^ Approximately, 1 in 5 patients who undergo protocol biopsy have subclinical alloimmune graft injuries that cannot be detected by noninvasive methods. Hence, the management of IS without histopathologic evaluation is challenging.^2,4^ Given the frequency of subclinical T cell–mediated rejection (subTCMR) in aLTR, a noninvasive test with a high negative predictive value could avoid liver biopsy in a relevant proportion of patients while providing safety in the management of IS.^4-6^

Recently, Zhang et al^7^ presented an elegant approach to detect a noninvasive marker for subTCMR in a cohort of pediatric liver transplant recipients (pLTRs). In short, they performed bulk RNA-seq from pLTR-derived graft tissue with subTCMR and non-subTCMR. After performing differential gene expression (DGE), protein–protein interaction network, and hub gene analysis, C-X-C motif chemokine ligand 8 (CXCL8) emerged as a possible target for the detection of subTCMR. They performed experimental verification using qPCR, Western blot and immunohistochemistry. Finally, they measured CXCL8 in serum from 138 pLTR and showed encouraging results with an area under the curve (AUC) of 0.97, sensitivity of 95%, and specificity of 94.6% for the detection of subTCMR. Such an accurate marker could replace protocol biopsies in the personalized management of IS without missing patients with subclinical inflammation who may benefit from maintaining or even increasing IS^.7^ Own previous data from aLTR allografts have shown significant DGE mainly in patients with clinical TCMR compared with no histologic evidence of rejection (NHR); however, CXCL8 was not individually evaluated.^5^

The primary aim of this study was to assess the accuracy of plasma CXCL8 concentrations in a cohort of aLTR for the detection of subTCMR and for the identification of aLTR within or outside the current histologic classification for IS reduction. To evaluate the CXCL8 graft expression in patients with subTCMR, a previously published cohort in which CXCL8 graft expression was measured as a part of a gene panel in aLTR with different rejection phenotypes was reanalyzed.^5^

## MATERIALS AND METHODS

This was a single-center, retrospective study. We included aLTR (>18 y) at the time of the liver biopsy without replicative viral hepatitis from our ongoing prospective biomaterial repository. Written informed consent was obtained in advance from all patients. This study was approved by the local ethics committee (protocol number 933 for project Z2 of Comprehensive Research Center 738; MHH Ethikkommission, Hannover, Germany). Blood samples were collected, if possible, on the day of liver biopsy, but not later than 24 h after the liver biopsy. EDTA plasma was cryopreserved at −80 °C. The samples that were included in this study were collected between 2009 and 2017. Two cohorts were evaluated. The first is a reanalysis of a previously published cohort in which liver tissue transcriptomics was assessed, but CXCL8 was not individually assessed.^5^ This was to determine gene expression within graft tissue as tissue was not available consistently in the second cohort. For the second cohort with available plasma samples, we measured plasma CXCL8 concentrations in patients with NHR, subTCMR, clinTCMR, and heterogeneous liver injury group including antibody-mediated rejection, metabolic-associated steatohepatitis, and indeterminate inflammation according to Banff.^3^

### Definitions and Histologic Evaluation

Histologic grading and classification were performed by experienced liver pathologists and classified according to current scores. Graft inflammation was graded according to the rejection activity index (RAI),^3^ modified Ishak histological activity index (mHAI),^8^ and the Banff Mini score for IS reduction (portal tract inflammation ≤ 1, interface hepatitis ≤ 1, central perivenulitis ≤ 1, lobular inflammation = 0, biliary inflammation = 0, endothelialitis = 0, portal microvasculitis = 0, and periportal fibrosis ≤ 3) from the 2016 Banff consensus.^3^ Graft fibrosis was assessed using the liver allograft fibrosis score^9^ and the Ishak fibrosis score (Ishak F).^8^

Significantly elevated liver enzymes were defined as aspartate aminotransferase, alanine aminotransferase, alkaline phosphatase ≥ 2 × upper limit of normal. NHR was defined as RAI score ≤1 + 0 + 0 (portal, biliary, and venous endothelial inflammation) in the absence of significantly elevated liver enzymes and fibrosis according to Ishak F < 2 or liver allograft fibrosis <2. SubTCMR was defined by a Banff RAI ≥1 + 1 + 1 in the absence of significantly elevated liver enzymes. ClinTCMR was defined by a Banff RAI ≥1 + 1 + 1 with significantly elevated enzymes. Significant fibrosis was defined as Ishak F ≥ 2.

### Plasma CXCL8 Measurement

Plasma CXCL8 concentrations were measured with a commercially available ELISA (Sigma Aldrich (RAB0319), Saint Louis, MO) according to the manufacturer’s instructions. Briefly, 100 μL of each standard and plasma sample (×2-fold dilution) were added to the wells and incubated for 2.5 h at room temperature. After washing, 100 μL of 1 × biotinylated detection antibody was added and incubated for 1 h. After a second wash, horseradish peroxidase-Streptavidin was applied and incubated for 45 min. Finally, a third wash was performed, TMB (3,3′,5,5′-tetramethylbenzidine) reagent was added, and the photometric reaction was stopped after 30 min. The optical density was measured in an ELISA reader (Tecan Sunrise-Basic, Grödig, Austria) at 450 nm. The lower quantitation limit (LOQ) for this ELISA is 0.8 pg/mL. For calculation purposes, samples that fell under the LOQ were substituted by the LOQ/2, that is, 0.4 pg/mL.

### Statistical Analysis

Statistical analysis was performed using R statistical software (version 4.1.2, R Core Team). The distribution of variables for demographic characteristics was assessed using the Shapiro–Wilk test. As all numerical variables had nonnormal distribution, all variables are presented as median and range, whereas categorical variables are presented as frequencies and percentages. Comparisons between groups were made using the Wilcoxon rank sum test for continuous variables and the Fisher exact test for categorical variables. When comparing 3 groups with numerical variables, post hoc analysis was performed when the Kruskal–Walli test indicated significant differences between groups, using the Dunn test adjusted by the Bonferroni method to determine individual differences. For categorical variables, adjusted Fisher exact test was used as a post hoc analysis.^10^ A *P* value of ≤0.05 (two-sided) was considered statistically significant. Correlations between continuous parameters were performed using Spearman correlation (Spearman ρ). The optimal cutoff was defined as the point maximizing the Youden function. Receiver operator characteristic curve and Youden index analysis were performed using the Cutpointr package.^11^ For the comparison of differentially expressed genes the p-values were adjusted according to the Bonferroni method.^12^

## RESULTS

As a first step to confirm the overexpression of CXCL8 in graft tissue from patients with subTCMR, we reanalyzed own previously published data from 77 aLTR (NHR: 25, subTCMR: 36, and clinTCMR: 16) in which the expression of 93 transcripts was assessed by qPCR.^5^ In these data, we found significant CXCL8 graft overexpression between patients with clinTCMR and NHR (*P* adj. < 0.01), but no difference in CXCL8 graft expression between patients with subTCMR and NHR (*P* adj. = 1.00; **Figure S1, SDC**, http://links.lww.com/TXD/A624).

For the CXCL8 plasma concentration measurement, 78 aLTRs were included: 18 (23.1%) patients with NHR, 11 (14.1%) patients with clinTCMR, 34 (43.6%) patients with subTCMR, and 15 (19.2%) patients with other conditions (4, possible chronic antibody-mediated rejection; 4, indeterminate for TCMR according to Banff criteria [RAI score = 2]; 3, steatotic liver disease; 2, unspecific hepatitis; 1 possible drug-induced liver injury; 1, moderate fibrosis). In the overall cohort, 49 (62.8%) patients were male, and the median age at transplantation was 48 [38.5–56.2] y. The most frequent indication for transplantation was autoimmune liver disease in 27 (34.6%) patients, followed by alcoholic liver disease in 25 (19.2%) patients. As the selected cohort was collected between 2009 and 2017, 37 (47.4%) patients were managed with cyclosporine as the primary IS therapy and 41 (52.6%) with tacrolimus.

Patients in the heterogeneous etiology group were older at the time of the liver biopsy than patients with subTCMR (57.0 [25.0–68.0] y versus 44.5 [21.0–65.0] y, *P* = 0.02), but no other differences in patients’ age at the time of liver biopsy were observed between the other groups (Table 1). No significant difference was observed between the groups with respect to etiology (Table 1). As expected, patients with clinTCMR had higher concentrations of liver transaminases and cholestatic markers according to the diagnostic criteria (Table 1). Also, as expected, patients with NHR had less inflammation and fibrosis in the histologic findings (Table 1). Regarding the IS management, patients with clinTCMR were mainly treated with cyclosporine (81.8%) and patients with NHR were mainly treated with tacrolimus (72.2%; Table 1).

**TABLE 1.** Characteristics of the cohort

	A. NHR	B. subTCMR	C. clinTCMR	D. Other	*P*	Post hoc *P*					
No.	18	34	11	15		AB	AC	AD	BC	BD	CD
Female sex	7 (38.9)	10 (29.4)	8 (72.7)	4 (26.7)	0.06						
Age at liver biopsy	52.5 [22.0–65.0]	44.5 [21.0–65.0]	48.0 [19.0–64.0]	57.0 [25.0–68.0]	**0.03**	0.52	1.00	0.52	1.00	**0.02**	0.39
Etiology					0.06						
Alcohol-related liver disease	4 (22.2)	3 (8.8)	2 (18.2)	2 (13.3)							
Autoimmune liver diseases	1 (5.6)	18 (52.9)	2 (18.2)	6 (40.0)							
Chronic viral hepatitis	6 (33.3)	3 (8.8)	1 (9.1)	5 (33.3)							
Cryptogenic liver disease	3 (16.7)	4 (11.8)	3 (27.3)	1 (6.7)							
Other	4 (22.2)	6 (17.6)	3 (27.3)	1 (6.7)							
Liver values											
AP × ULN	0.6 [0.2–1.1]	0.7 [0.2–1.3]	2.5 [1.0–8.9]	0.9 [0.3–1.3]	**<0.01**	0.14	**<0.01**	0.05	**<0.01**	0.33	**<0.01**
ALT × ULN	0.5 [0.2–1.4]	0.5 [0.2–1.8]	5.0 [1.6–11.8]	0.4 [0.3–0.7]	**<0.01**	1.00	**<0.01**	1.00	**<0.01**	1.00	**<0.01**
AST × ULN	0.7 [0.4–1.7]	0.7 [0.4–1.5]	4.5 [1.5–15.3]	0.8 [0.5–1.0]	**<0.01**	1.00	**<0.01**	1.00	**<0.01**	1.00	**<0.01**
gGT × ULN	0.4 [0.1–1.8]	0.5 [0.2–4.3]	8.6 [1.3–38.7]	0.7 [0.3–1.9]	**<0.01**	0.48	**<0.01**	0.17	**<0.01**	0.48	**<0.01**
Immunosuppression											
Prednisolone	11 (61.1)	29 (85.3)	9 (81.8)	10 (66.7)	0.20						
Tacrolimus	13 (72.2)	20 (58.8)	2 (18.2)	6 (40.0)	**0.02**	0.39	**0.047**	0.17	0.11	0.39	0.39
Cyclosporin	5 (27.8)	14 (41.2)	9 (81.8)	9 (60.0)	**0.02**	0.39	**0.047**	0.17	0.11	0.39	0.39
mTOR inhibitor	1 (5.6)	1 (2.9)	0 (0.0)	0 (0.0)	0.72						
Mycophenolate mofetil	14 (77.8)	33 (97.1)	9 (81.8)	15 (100.0)	0.05						
Histological findings											
RAI total score	1.0 [0.0–1.0]	3.5 [3.0–6.0]	5.0 [3.0–5.0]	3.0 [1.0–5.0]	**<0.01**	**<0.01**	**<0.01**	**<0.01**	0.24	0.24	0.05
Ishak mHAI score	1.0 [0.0–2.0]	3.0 [1.0–6.0]	5.0 [2.0–8.0]	3.0 [1.0–7.0]	**<0.01**	**<0.01**	**<0.01**	**<0.01**	**0.01**	0.26	0.26
Ishak F	0.0 [0.0–1.0]	1.0 [0.0–4.0]	1.0 [0.0–3.0]	1.0 [0.0–4.0]	**<0.01**	**<0.01**	**<0.01**	**0.01**	1.00	1.00	1.00
Total LAF	0.0 [0.0–3.0]	1.0 [0.0–8.0]	2.0 [0.0–4.0]	2.0 [0.0–7.0]	**<0.01**	**0.01**	**<0.01**	**0.01**	0.77	1.00	1.00
Months between LT and biopsy	12.0 [5.0–59.0]	23.0 [5.0–94.0]	16.0 [2.0–46.0]	38.0 [12.0–70.0]	**0.01**	0.18	0.73	**<0.01**	0.72	0.18	0.09
Plasma CXCL8 (pg/mL)	3.6 [0.4–22.0]	11.5 [0.4–41.0]	9.4 [0.4–40.5]	8.7 [0.4–31.2]	0.24						

Data are provided as n (%) or median [range]. Post hoc analysis was performed with Dunn test adjusted by the Bonferroni for continuous variables and Fisher exact test for independence for categorical variables.

Bold indicates statistically significant *P* value of ≤0.05 (two sided).

AP, alkaline phosphatase; ALT, alanine aminotransferase; AST, aspartate aminotransferase; clinTCMR, clinical T cell–mediated rejection; gGT, gamma glutamyl-transpeptidase; Ishak F, Ishak fibrosis score; LAF score, liver allograft fibrosis score according to Venturi et al^9^; LT, liver tranplant; mHAI, modified histological activity index according to Ishak et al^8^; mTOR, mechanistic target of rapamycin; NHR, nonhistological rejection; RAI, rejection activity index^3^; subTCMR, subclinical T cell–mediated rejection; ULN, upper limit of normal.

**FIGURE 1.**
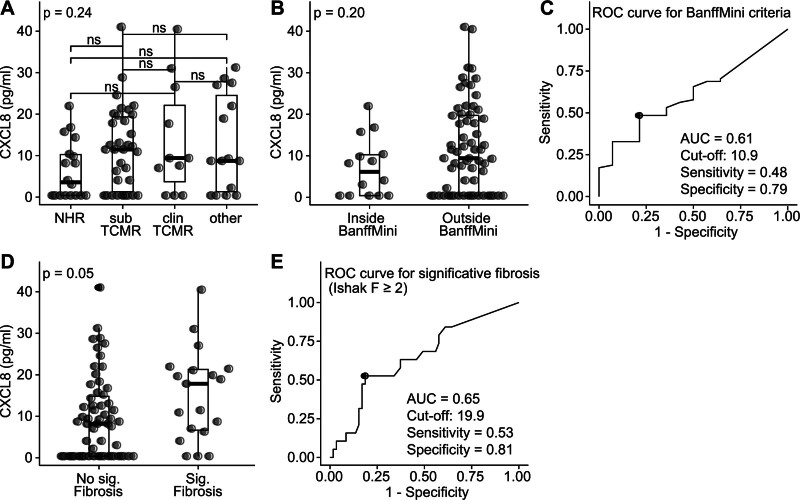
Plasma CXCL8 concentrations in different groups and ROC curves for reduction of immunosuppression and fibrosis. A, Plasma CXCL8 (pg/mL) concentrations in patients with NHR, subTCMR, clinTCMR and patients with other etiologies. B, Plasma CXCL8 (pg/mL) concentrations in patients within and outside of the histological criteria for immunosuppression reduction (Banff Mini criteria).^3^ C, ROC curve, sensitivity, specificity, and optimal cutoff concentration for the detection of patients outside of the Banff Mini criteria. D, Plasma CXCL8 (pg/mL) concentrations of patients with (Ishak F ≥ 2) and without significant fibrosis (Ishak F < 2). E, ROC curve, sensitivity, specificity, and optimal cutoff concentration for the detection of patients with significant fibrosis (Ishak F ≥ 2). AUC, area under the curve; clinTCMR, clinical T cell–mediated rejection; CXCL8, C-X-C motif chemokine ligand 8; Ishak F, Ishak fibrosis score; NHR, nonhistological rejection; ROC, receiver operator characteristic; subTCMR, subclinical T cell–mediated rejection.

**FIGURE 2.**
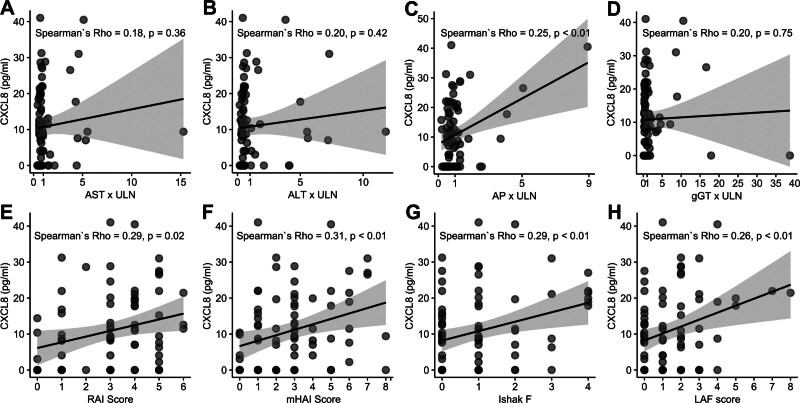
Correlation between CXCL8 and, liver chemistry values and histologic scores. A–D, Correlation with linear regression and Spearman rank correlation (Spearman ρ) between plasma CXCL8 (pg/mL) concentrations, and liver transaminases (ALT and AST) × ULN and cholestatic markers (AP and gGT) × ULN. E–H, Correlation with linear regression and Spearman rank correlation (Spearman ρ) between plasma CXCL8 (pg/mL) concentrations, and histological inflammation scores (RAI score and mHAI score), and histological fibrosis scores (Ishak F and LAF scores). ALT, alanine aminotransferase; AP, alkaline phosphatase; AST, aspartate aminotransferase; CXCL8, C-X-C motif chemokine ligand 8; gGT, gamma glutamyl-transpeptidase; Ishak F, Ishak fibrosis score; LAF, liver allograft fibrosis^9^; mHAI, modified Ishak modified histological activity index^8^; RAI, rejection activity index^3^; Spearman ρ, Spearman rank correlation; ULN, upper limit of normal.

No significant differences in median plasma CXCL8 (pg/mL) concentrations were observed between the groups (Figure 1A). Patients with NHR had median CXCL8 levels of 3.6 [0.4–22.0] pg/mL, patients with subTCMR had median levels of 11.5 [0.4–41.0] pg/mL, patients with clinTCMR had median levels of 9.4 [0.4–40.5] pg/mL, and patients with other etiologies had median levels of 8.7 [0.4–31.2] pg/mL (Table 1; Figure 1A). Plasma CXCL8 concentrations did not correlate with sample storage time (**Figure 2, SDC**, http://links.lww.com/TXD/A625).

Regarding the capacity of CXCL8 to discriminate between patients with (n = 14) or without (n = 64) relevant subclinical graft injury according to the Banff Mini criteria for IS reduction, no differences were observed between the two groups (Figure 1B). The sensitivity, specificity, and optimal cutoff value of CXCL8 for the detection of patients outside the BanffMini criteria were calculated. The optimal cutoff using the maximum Youden index was 10.9 pg/mL, with a sensitivity of 0.48, specificity of 0.79, and AUC of 0.61 (Figure 1C).

Only negligible to weak correlations were found between CXCL8 concentrations and liver chemistry values (Spearman ρ = 0.18–0.25; Figure 2A–D) and CXCL8 concentrations and histological inflammation or fibrosis scores (Spearman ρ = 0.26–0.31; Figure 2E–H). However, a significant difference in plasma CXCL8 levels was observed when patients were classified as having significant (17.8 [0.4–40.5] pg/mL) or nonsignificant fibrosis (8.2 [0.4–41.0] pg/mL; Figure 1D). The sensitivity, specificity, and optimal cutoff value of CXCL8 for the detection of patients with significant fibrosis were also calculated, and the optimal cutoff using the maximum Youden index was 17.8 pg/mL, with a sensitivity of 0.53, specificity of 0.81, and AUC of 0.65 (Figure 1E).

## DISCUSSION

In this article, we explored the use of plasma CXCL8 concentrations for the identification of subTCMR as well as for the identification of patients within or outside the Banff Mini histologic criteria for IS reduction in aLTR as a noninvasive alternative to protocol biopsies in analogy to a most recent report in pLTR. Unfortunately, we could not replicate the recently reported results from pTLR that demonstrated CXCL8 as an accurate marker for the detection of subTCMR.^7^ We did not observe any statistical difference in the plasma CXCL8 concentrations between patients with NHR, subTCMR, clinTCMR, and other causes of graft injury. In addition, the sensitivity and specificity for classifying patients who could benefit from IS reduction according to the histologic criteria was low.

CXCL8 (formerly known as interleukin-8) is perhaps the most potent neutrophil-attracting chemokine and is produced not only by leukocytes but also by almost any cell that responds to inflammatory stimuli, including hepatocytes.^13,14^ In addition to its neutrophil chemoattractant activity, CXCL8 is a potent inducer of smooth muscle and primary hepatic stellate cells and plays an important role in the development of fibrosis.^13-15^ This is in line with our findings that patients with significant fibrosis had borderline higher concentrations of plasma CXCL8. However, the high overlap between the groups, resulting in low sensitivity and specificity, rules out this marker as a noninvasive marker of fibrosis and just recently, markers that quantify extracellular matrix turnover have demonstrated to exhibit higher AUCs for the noninvasive detection of significant graft fibrosis in aLTR.^16^ In addition, liver stiffness measurements have demonstrated excellent accuracy to detect significant fibrosis in aLTR further limiting the potential of CXCL8 as a noninvasive marker for the detection of fibrosis.^17^ Higher CXCL8 concentrations have also been reported in a variety of liver inflammatory conditions as well as nonliver inflammatory conditions, for example, severe sepsis or cancer, and are not specific for an alloimmune response, including chronic liver diseases, such as alcoholic liver disease, cholestatic liver diseases, and chronic hepatitis C infection.^13-15,18,19^ Serum CXCL8 concentrations in the context of chronic liver diseases have been closely linked to cirrhosis, in a previous study with patients with chronic liver disease, and no cirrhosis had CXCL8 serum concentrations comparable to healthy individuals which further supports the association of CXCL8 with fibrosis in our study rather than subclinical inflammation.^15^ In the adult liver transplantation field, 1 prospective study analyzed CXCL8 concentrations in 317 samples from 19 patients. In this study, higher CXCL8 concentrations were associated with infectious complications and alloimmune rejection (6 cases of clinTCMR, in which 4 patients had elevated CXCL8 concentrations [mean = 76 ± 26 pg/mL]), whereas in patients without infectious complications or rejection, CXCL8 concentrations were below the LOQ. CXCL8 was considered a marker of inflammation rather than a specific marker for rejection with reported levels in the range of our study.^20^ Protocol biopsies were not performed in this study, and to our knowledge there are no previous reports in aLTR analyzing the utility of plasma CXCL8 for the diagnosis of subclinical alloimmune injury.

The contrast between our results and those published by Zhang et al^7^ may be due to the fact that aLTR have a different alloimmune milieu than pLTR, a greater variety of possible etiologies causing liver disease, the nature of complications, and a different immunologic environment. The complexity of the immunological response in graft rejection and the overlap with nonalloimmune inflammatory conditions make it difficult to identify single markers that could detect the presence of subclinical alloimmune injury with both high sensitivity and specificity, at least in aLTR. However, it is important to recognize the approach taken by Zhang et al,^7^ in which a highly accurate serum marker for subTCMR in pLTR was found from tissue bulk RNA-seq data through experimental verification with Western blot and tissue immunohistochemistry. This was a remarkable approach that could be taken as a model for the development of novel markers for rejection after transplantation. However, it is important to emphasize that the immunologic milieu in the blood is not always representative of that in the graft,^21^ making it difficult to translate tissue markers to blood markers. Although considerable overlap of the T-cell receptor repertoire was demonstrated in acute rejection in aLTR, smoldering alloimmune injury did not lead to alignment of peripheral and central T-cell milieu adding another possible explanation for our findings.^21^

Regarding the expression of the graft transcriptome in patients with subTCMR, there are already data in aLTR that demonstrate the DGE between NHR and subTCMR not to be as significant as in the case of clinTCMR.^22^ Moreover, the reanalysis of these data confirmed that, in aLTR, there is no tissue overexpression of CXCL8 in patients with subTCMR compared with patients with NHR. In addition, CXCL8 concentrations in our cohort are similar to those reported in healthy patients (31.83 ± 81.8 pg/mL),^23^ which is also consistent with the findings of our DGE data. Other studies using the same commercial ELISA (Sigma Aldrich) have reported CXCL8 concentrations of 57.0 ± 11.0 in healthy individuals.^24^

We recognize the limitations of this brief report, namely the retrospective nature of the study, and the small size of the cohort, with only 34 patients with subTCMR, and 44 patients with other conditions. Furthermore, there are 3 characteristics of the cohort that are important to highlight: A heterogenous sampling time point, between 2 and 94 mo, after transplantation; a high proportion of autoimmune liver diseases in the subTCMR group, although not significantly different from the other groups; and finally regarding the IS, a high use of steroids, which reduce cytokine production in cell culture experiments,^25^ and high use of cyclosporine therapy in almost half of the patients is not the current standard of care. However, a reliable marker for the diagnosis of rejection should be helpful for all aLTR regardless of the time after transplantation, the etiology that led to the transplantation and the IS management. Finally, some of the samples selected for this study have been stored since 2009, which, even when stored at −80 °C, carries a risk of cytokine degradation^26^; however, we saw no inverse correlation between the year of sampling and the plasma CXCL8 concentrations (**Figure S2, SDC**, http://links.lww.com/TXD/A625). In addition, there are no data that investigate if the procedure of the liver biopsy affects levels of CXCL8 in serum or plasma. For our samples, we did not capture if the blood drawing was done before or after the puncture. Hence, we cannot address this potential source of bias.

Overall, the results presented, including the gene expression data and plasma CXCL8 concentrations, and the previously published data showing elevated CXCL8 in other inflammatory nonalloimmune liver diseases, as well as nonliver inflammatory conditions, currently preclude the use of plasma CXCL8 quantification to noninvasively detect subclinical liver injury in aLTR with further studies being needed. In conclusion, plasma CXCL8 appears not to detect subclinical alloimmune injury as well as the histologic criteria for IS reduction in aLTR with a high-accuracy cross sectionally.

## Supplementary Material


