# Supplementary figures and images for: Elevated Plasma CXCL8 Concentrations in Significant Fibrosis but Not in Subclinical Rejection After Adult Liver Transplantation
Source: Transplant Direct. 2024 Feb 21;10(3):e1592. doi: 10.1097/TXD.0000000000001592 (PMC11774564; doi:10.1097/TXD.0000000000001592)

CXCL8 Liver tissue Expression(delta-delta CT)

$p = 1.00$

$p = 0.18$

$p < 0.01$

NHR

subTCMR

clinTCMR

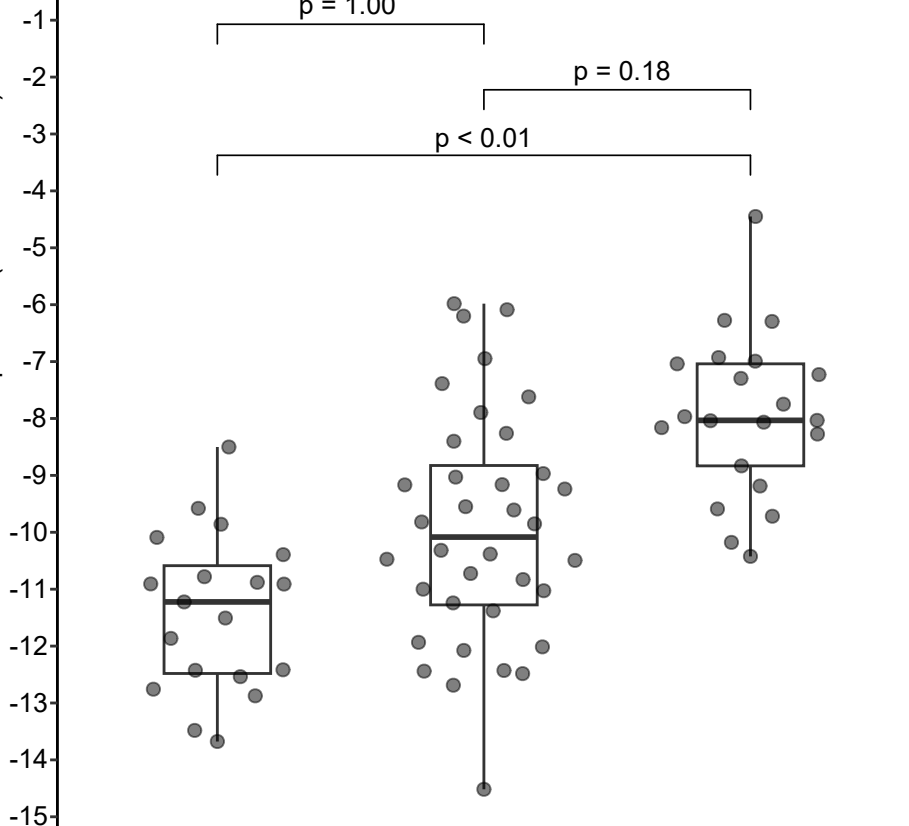

Supplement: Supplementary file 1 [file txd-10-e1592-s001.pdf]

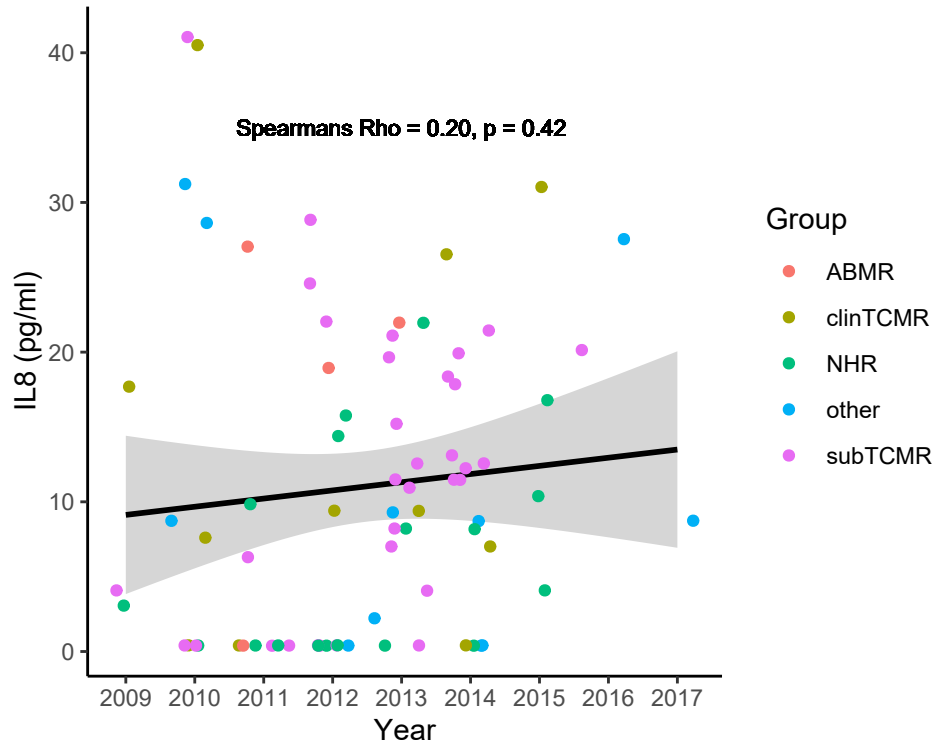

Supplement: Supplementary file 2 [file txd-10-e1592-s002.pdf]
